# Development and Evaluation of an Automated Manual Resuscitator-Based Emergency Ventilator-Alternative

**DOI:** 10.7759/cureus.13642

**Published:** 2021-03-01

**Authors:** Jesica Urbina, Stormy M Monks, Luis Ochoa, Robert F Stump, Ryan B Wicker, Chris Danek, Victor I Torres, Scott B Crawford

**Affiliations:** 1 Training and Educational Center for Healthcare Simulation (TECHS), Texas Tech University Health Sciences Center El Paso, El Paso, USA; 2 W.M. Keck Center for 3D Innovation, The University of Texas at El Paso, El Paso, USA; 3 Emergency Medicine, Texas Tech University Health Sciences Center El Paso, El Paso, USA; 4 Regulatory Affairs, Bessel LLC, San Carlos, USA; 5 Emergency Medicine; Training and Educational Center for Healthcare Simulation (TECHS), Texas Tech University Health Sciences Center El Paso, El Paso, USA

**Keywords:** automated bvm compression device, bag valve mask, emergency ventilator, manual resuscitator, mechanical ventilator

## Abstract

Mass casualty incidents such as those that are being experienced during the novel coronavirus disease (COVID-19) pandemic can overwhelm local healthcare systems, where the number of casualties exceeds local resources and capabilities in a short period of time. The influx of patients with lung function deterioration as a result of COVID-19 has strained traditional ventilator supplies. To bridge the gap during ventilator shortages and to help clinicians triage patients, manual resuscitator devices can be used to deliver respirations to a patient requiring breathing support. Bag-valve mask (BVM) devices are ubiquitous in ambulances and healthcare environments, however require a medical professional to be present and constantly applying compression to provide the patient with respirations.

We developed an automated manual resuscitator-based emergency ventilator-alternative (AMREV) that provides automated compressions of a BVM in a repetitive manner and is broadly compatible with commercially-available BVM devices approximately 5 inches (128 mm) in diameter. The AMREV device relieves the medical professional from providing manual breathing support and allows for hands-free operation of the BVM. The AMREV supports the following treatment parameters: 1) adjustable tidal volume (V*_T_*), 2) positive end-expiratory pressure (PEEP) (intrinsic and/or external), 3) 1:1 inspiratory: expiratory ratio, and 4) a controllable respiratory rate between 10-30 breaths per minute.

The relationship between the inherent resistance and compliance of the lung and the delivered breaths was assessed for the AMREV device. Adjustable V*_T_* of 110-700 ml was achieved within the range of simulated lung states. A linear increase in mean airway pressure (P*_aw_*), from 10-40 cmH_2_O was observed, as the resistance and compliance on the lung model moved from normal to severe simulated disease states. The AMREV functioned continuously for seven days with less than 3.2% variation in delivered V*_T_* and P*_aw_*. Additionally, the AMREV device was compatible with seven commercially-available BVM setups and delivered consistent V*_T_* and P*_aw_* within 10% between models.

This automated BVM-based emergency-use resuscitator can provide consistent positive pressure, volume-controlled ventilation over an extended duration when a traditional ventilator is not available. True ventilator shortages may lead to manual resuscitators devices such as the AMREV being the only option for some healthcare systems during the COVID-19 pandemic.

## Introduction

In late December 2019, an outbreak of a novel coronavirus disease (COVID-19) was reported in Wuhan, China, and was subsequently identified as a pandemic [1]. Severe COVID-19 disease can result in death due to the development of an acute respiratory distress syndrome (ARDS)-like illness [2]. Invasive ventilation is indicated for adult patients with ARDS-associated respiratory difficulty [3, 4]; however, the influx of patients with lung function deterioration as a result of COVID-19 has strained traditional ventilator supplies [1, 5-7]. To bridge the gap during ventilator shortages, automated manual resuscitator devices can be used to deliver respirations to a patient requiring breathing support [8].

There are currently many open-source solutions that seek to disseminate manual resuscitator (bag-valve mask; BVM) ventilator designs with the hope of bridging the gap during the COVID-19 pandemic [9-11]. However, there are important design considerations when using BVM devices for this type of off-label use to prevent further lung injury from barotrauma [12]. First, the design should ensure that the device does not degrade the BVM performance with regard to pressure, V*_T,_* and flow waveforms from repetitive device motion. Durability testing has to be performed not only on the mechanical system but also on the BVM device. Secondly, even with a fixed BVM compression setting, the V*_T_* delivered is dependent on the resistance of the airway and the compliance of the lungs [13]. The only respiratory parameter that can be controlled by most devices is the respiratory rate, and while the United States (U.S.) Food and Drug Administration (FDA) has issued emergency use authorizations for devices in which V*_T_* can be selected [14], designs with a fixed stroke length (compression setting) lead to inconsistent V*_T_* delivered as pulmonary parameters change. Ventilator designs should measure expired volumes or predict V*_T_* across ventilator settings for specific pulmonary resistance and compliance parameters.

For this work, it was hypothesized that a low-cost, emergency-use automated BVM-based device could consistently deliver mechanical respirations with a variety of widely commercially-available BVMs. The specific aims were to 1) rapidly design and develop a volume-controlled automated manual resuscitator-based emergency ventilator-alternative with user-adjustable volume and respiratory rate, 2) evaluate the parameters of V*_T_*, rate, and mean airway pressure over different resistance and compliance settings that would be representative of patients with varying lungs states, and 3) establish longevity and implementation of the device across multiple BVM models.

The designed device is a low-cost emergency ventilator-alternative with adjustable stroke length and a housing enclosure that was developed to constrain BVM deformation in a reliable manner to ensure consistent V*_T_* is delivered for each pulmonary parameter. The device, an automated manual resuscitator-based emergency ventilator-alternative (AMREV), was assessed for V*_T_* delivered, mean airway pressure (P*_aw_*), and respiratory rate for different simulated models of lung disease. To ensure that variation among commercially-available BVMs did not create the risk of going over threshold V*_T_* or P*_aw_*, design considerations were made to ensure that the ventilator device would be widely compatible with BVM models measuring up to approximately 128 mm (5 inches) in diameter, with a 1.5 L air reservoir.

## Materials and methods

A. Design and development of a volume-controlled manual resuscitator-based emergency ventilator

**Figure 1:**
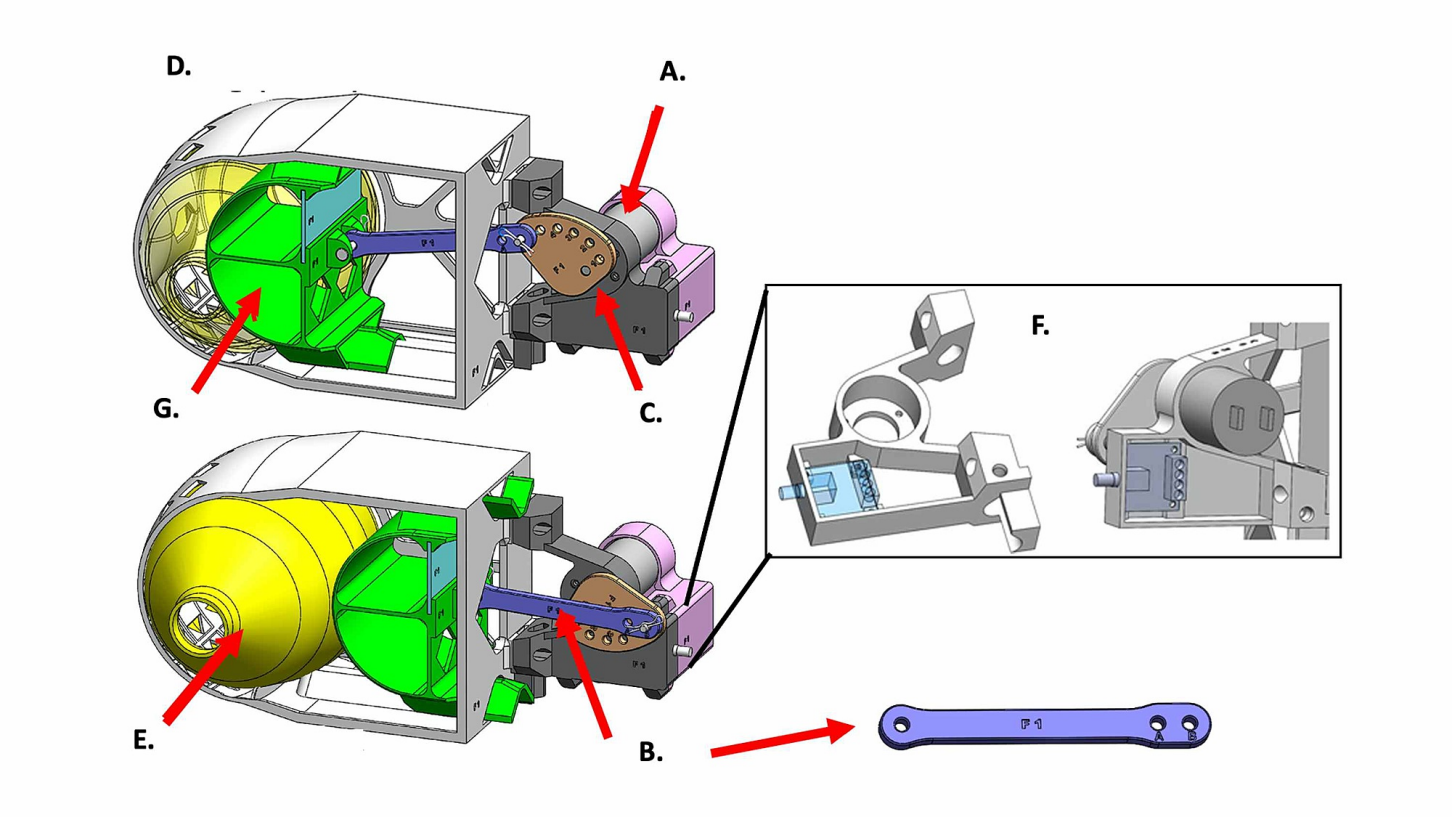
Schematic of AMREV, automated manual-resuscitator based emergency ventilator-alternative A) Motor; B) Piston rod; C) Cam; D) Housing; E) Bag-valve mask; F) Motor control enclosure; G) Piston.

The AMREV was created using additive manufacturing (hereafter referred to as 3D-printing) technology combined with traditional build techniques that allowed for rapid design iterations and assessments. The design uses an eccentric attachment of a rod and piston assembly to a cam plate driven by a simple low-voltage geared motor to compress the BVM (Figure. 1). The rod attachment point on the cam controls the amount of compression of the manual resuscitator bag and therefore V*_T_*. Respiratory rate of the 30 rpm (without a load) geared motor is controlled by a hardware-based pulse-width modulation (PWM) controller. Full user assembly and operation instructions are included in the supplemental materials Appendix A 'Instructions for Use'.

The 3D-printed components of the device were made of polycarbonate material fabricated on Stratasys 400 mc, 450 mc, and 900 mc Fortus series printers (Rehovot, Israel). Polycarbonate was selected due to its strength, availability, durability, and acceptability in medical applications. Electrical power to the AMREV unit was provided by a commercially-available low-voltage 12V 2A DC power supply. Electrical connections were made within the enclosed 3D printed motor mount, and secured using screw terminals, solder, and commercial spade clips. Hitch pin clips were used to secure the clevis pins of the mechanical piston rod, a set screw is used to attach the adjustable-length cam to the motor shaft, and screws hold the electronics cover to the motor mount.

A rapid, iterative design/simulate, build, and test approach was used to converge on the final design. The main design considerations included 1) compatibility with commercial BVMs, 2) a piston-cylinder and sliding mechanism approach for bag compression, 3) device conforming to BVM shape to reduce stress, 4) minimizing weight, 5) minimizing ventilation dead space volume by allowing mounting near the patient’s mouth using a bedside IV pole, 6) an electric motor needed to drive the mechanism, and 7) compatibility with additive manufacturing (AM); specifically, fused deposition modeling (FDM) with polycarbonate material. Dimensions were selected during the design phase to ensure ease of assembly, operation on a sliding contact mechanism, and the specified range of piston travel. All were achieved by accounting for tolerances of the FDM process. To reduce variability in the manufacturing process and in device performance, the design excluded the use of support structures on the critical piston linear slide or BVM contact surfaces. Solid modeling was performed in Solidworks (Version 2019-20). Prototyping by 3D printing allowed rapid production and advancement through six major design iterations. A future publication will discuss the use of simulation by finite element analysis (FEA) in parallel with the AMREV design iterations as a precursor to device-life testing.

B. Tidal volume and pressure assessment

Prototypes were assessed for general function, piston movement, and length of piston travel needed to compress the manual resuscitator to deliver an approximate tidal volume between 200-700 ml in a patient with normal lung function with resistance 5 cmH_2_O.L^-1^.s^-1^ and compliance 50 ml.cmH_2_O^-1^. Device assessments were performed with a RespiTrainer Advance™ and QuickLung® (IngMar Medical Ltd., Pittsburg, PA, USA). After functional confirmation, specific parameter testing and device calibration was performed using an ASL 5000™ Breathing Simulator. (IngMar Medical Ltd., Pittsburg, PA, USA). The same IngMar® ASL 5000 was used for all testing parameters. Following data collection, the ASL 5000 was connected to a hospital-certified ventilator to compare calibration of V*_T_* and pressures. The device was found to correlate within 5% across assessed parameters.

To measure the effect of patient lung resistance and compliance on V*_T_* delivered, variation was assessed across different pulmonary parameter models. The initial disease states for testing were chosen based on discussions with a pulmonologist\critical care physician at our local county hospital about expected pulmonary parameters based on early COVID-19 patient information available. The simulated lung models used during testing had predefined resistance (R) (cmH2O.L-1.s-1) and compliance (C) (ml.cmH2O-1) parameters and were labeled ‘normal’ (R5C50), ‘mild disease’ (R6C20) and, 'moderate disease’ (R10C10) (see page 2 of the IngMar product sheet here (https://pdf.medicalexpo.com/pdf/ingmar-medical/quicklung-precision-test-lung/68861-105019.html). Additionally, a ‘severe disease’ state (R50C10) was tested for 24 hours to meet FDA and international standard requirements for basic safety and essential performance of ventilatory support (ISO 80601-2-80:2018; 201.104) [15, 16]. All four simulated pulmonary parameters were defined in the RespiSim® software (Version 3.6) to have zero muscle effort to simulate a paralyzed patient.

A single assembled unit with a 30 rpm motor, run at 100% power was paired with an adult Rüsch® (Wayne, PA, USA) BVM to assess device capability to meet ventilation requirements of patients with ARDS. An AirLife™ viral filter (Vyaire Medical, IL, USA) was attached to the BVM and connected to the ASL 5000 using the supplied five feet coil wrapped air hose. Sequential testing was performed across all connection parameters; rod positions A and B, with cam positions one to six (position A-six is not allowed due to the travel distance of the piston in the housing).

A minimum set of data for ten breaths were recorded within the RespiSim® software for analysis. Each stroke length and power level test was repeated using each of the three simulated patient parameters (normal, mild, and moderate lung disease). The RespiSim® software paired with the ASL 5000 breathing simulator recorded the variables of respiratory rate, Paw (cmH_2_O), inspiratory to expiratory (I:E) ratio, and V*_T_* (ml) for each test.

C. Longevity and implementation

**Figure 2:**
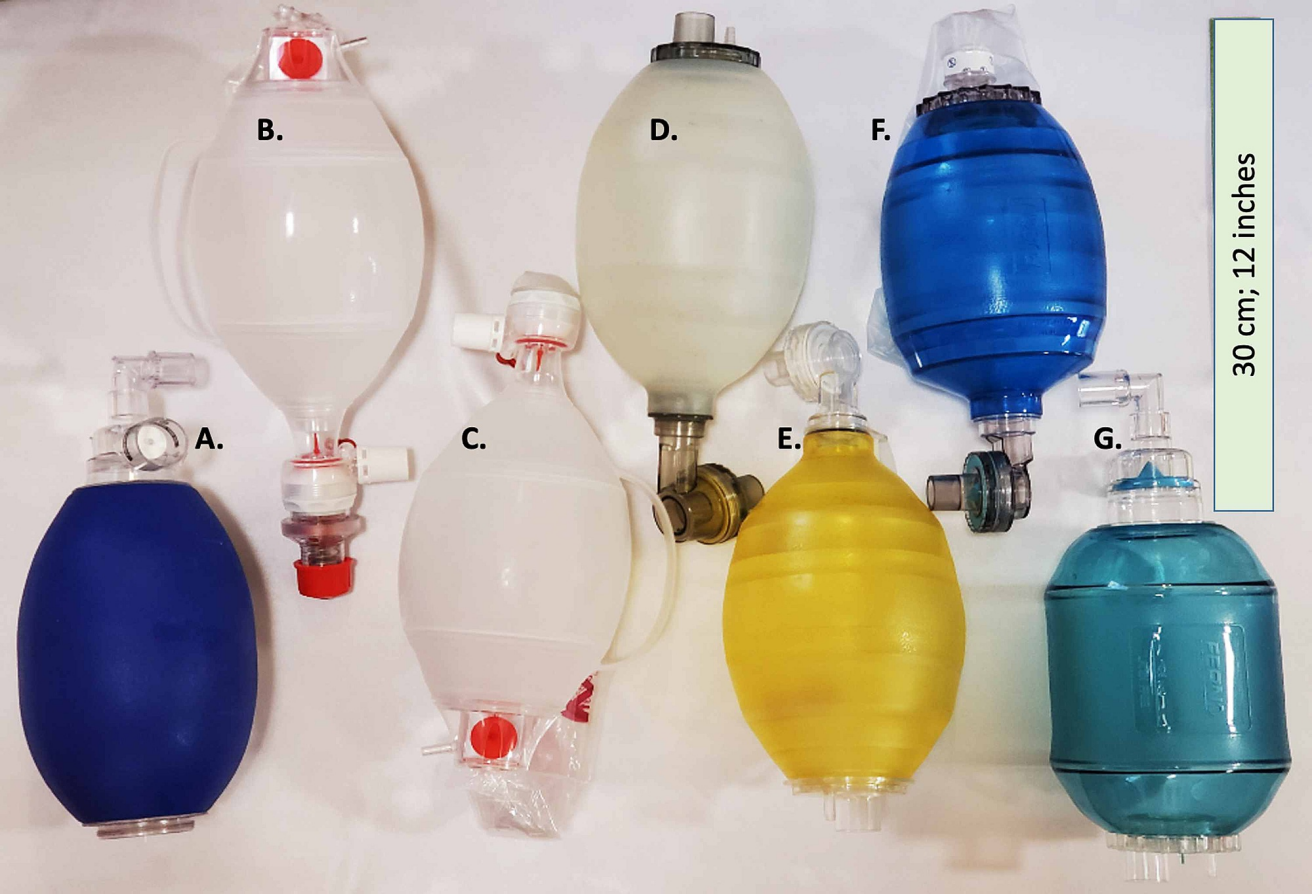
Manual resuscitator (BVM) models assessed for compatibility with AMREV. Shown from left to right (A-G) in descending order in Table 1.

Ventilatory parameters for seven BVMs representing six commonly-used, commercially-available models and manufacturers were assessed in conjunction with the AMREV (Figure 2, Table 1). Materials varied by brand and model and were one of three materials: silicone, polyvinyl chloride (PVC), or styrene-ethylene-butylene-styrene (SEBS) polymer [17-22]. For comparison testing, each bag was paired with a single AMREV device housing enclosing a 30 rpm motor, run at 75% power, with rod-cam position combination A-4 (stroke length 6.4 cm). Parameters of V*_T _*(ml) and pressure (cmH_2_O) for each breath were recorded by the ASL 5000 RespiSim software and exported for review. During longevity testing, each BVM was connected to an Ingmar Quicklung™ with settings of compliance 50 ml.cmH_2_O^-1 ^and resistance 5 cmH_2_O.L^-1^.s^-1^. Each BVM was temporarily disconnected from the Quicklung™ device for data collection with the ASL 5000 breathing simulator.

Means and standard deviations were calculated for P*_aw_* and V*_T_* across 13 breaths. The V*_T_* and P*_aw_* delivered at a single connection setting were compared between seven BVM setups for each of four disease states. Longitudinal comparisons of P*_aw_* and V*_T_* were also collected and analyzed at day zero and day seven for five BVM setups. Additionally, a single Rüsch BVM was also tested at 30 and 45 days to evaluate the consistency of P*_aw_* and V*_T_* delivered after approximately 1.3 million compressions. Data was not recorded on one Carefusion BVM setup due to a component failure on the AMREV on the seventh day of continuous operation.

**Table 1:** Brand and model of the BVM devices tested with listed material from manufacturer product information [17-22]. PEEP - positive end-expiratory pressure

	Brand	Model	Material	Bag diameter (mm; in)
A	Carefusion	AirLife^®^	PVC	128.5 [5.06]
B	Ambu	AMBU^®^ Bag SPUR^®^ II	SEBS	124 [4.88]
C	Ambu	AMBU^®^ Bag SPUR^®^ II w/ PEEP valve	SEBS	124 [4.88]
D	Laerdal	Laerdal LSR Reusable Resuscitator	Silicone	127 [5.0]
E	Laerdal	Laerdal The Bag II	PVC	128 [5.08]
F	Rüsch	Resuscitator BVM	PVC	122 [4.80]
G	Ferno	Adult manual resuscitator	PVC	126 [4.96]

## Results

A. Development of an automated volume-controlled BVM-based emergency ventilator-alternative

The AMREV was designed as a rapidly-deployable, hands-free automated manual resuscitator-based compression device that delivers respirations when there is no other ventilator alternative (Figure 1).

**Figure 3:**
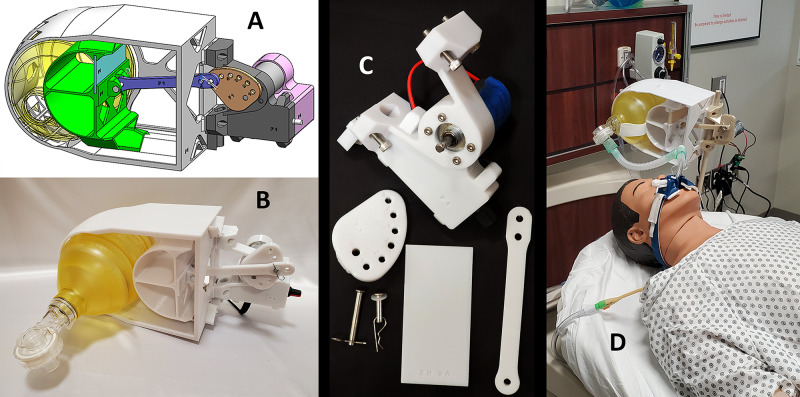
Design and function of the 3D-printed AMREV. A) Schematic for the device, B-C) 3D-printed parts, and D) fully assembled prototype device shown on a Laerdal MegaCode Kelly™ simulation manikin.

Consistent V*_T_* was delivered with six commercially-available BVMs trialed with the AMREV at each user selectable setting (Figure 2). The attachment points on the cam (Figure 1C) are labeled number one to six, with varying distances from the center and determine the travel distance of the piston rod and therefore, the V_T_ delivered with each breath. Each hole on the cam combined with the piston rod attachment point (A or B; Figure 1B) corresponds to a volume increment of approximately 50 - 110 ml depending on patient lung parameters. The piston rod is attached to a curved piston inside of the device housing enclosure (Figure 1D-G) that travels along a triangular-shaped linear slide to maintain smooth and directed operation toward a fixed wall. The BVM (Figure 1E) is placed between the piston and the wall, and with each cycle, the motor compresses the device to deliver a breath. The wall of the housing is curved to match the shape of the BVM. Return of the piston to its non-compressed state allows the bag to recoil and draw in oxygen-supplemented air. The BVM allows the patient to exhale through a one-way expiratory valve. After recoil, the BVM in the device is prepared to deliver another breath. There are 11 possible combinations between the attachment points A and B on the piston rod and the cam (A one to five; B one to six) (Figure 1B-C). Different combinations correspond to differing degrees of bag compression by the piston. The design uses a low-voltage, high-torque geared motor (Figure 1A) with an adjustable cycle of up to 30 rpm to drive an adjustable-length piston rod (Figure 1B) that is attached to a cam (Figur 1C) on the motor shaft. The user can adjust the motor rotation rate (breathing rate) by turning a knob on the outside of the motor casing (Figure 1F). Parts and implemented design are shown in Figure 3.

B. Tidal volume and pressure assessment for representative patient models with varying degree of lung function

Each specific setting of the rod and cam position selected by the user, corresponds to the degree of compression for the BVM, however, the actual V*_T_* delivered depends on patient lung resistance and compliance. To measure the effect of different pulmonary disease states on delivered volume, V*_T_* was assessed across assigned lung parameters. Respiratory rate was controlled by a 30 rpm motor operated at full power. The average breaths delivered per minute (bpm) for all 11 configurations was 27.6 ± 1.2 bpm. No variations in respiratory rate were observed across these lung states.

**Figure 4:**
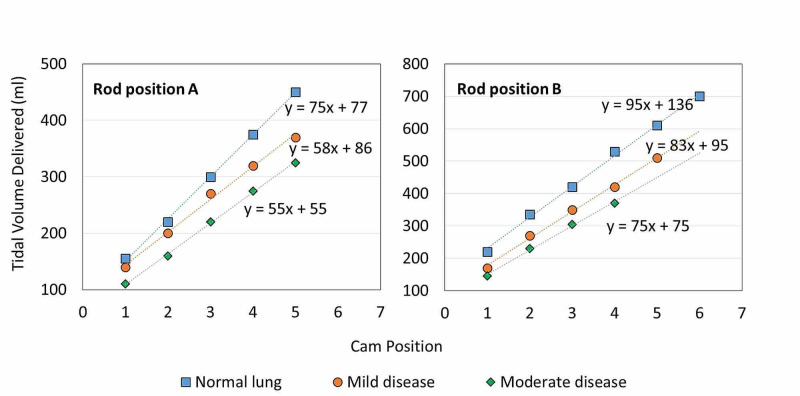
Tidal volume delivered for simulated patient types for rod settings A and B and cam settings 1-6.

In a simulated patient with normal lung function (R5C50), V*_T_* increased by 74 ± 6 ml for each change in cam settings one to five on piston rod position A and 91 ± 13 ml for cam settings one to six using piston rod position B (Figure 4, Table 2). As resistance of the lungs increased and compliance decreased, a decline in V*_T_* was observed within the same rod and cam settings. In the mild (R6C20) simulated lung disease model, V*_T_* increased in correspondence with changes in cam settings. Changes were 58 ± 10 ml and 85 ± 13 ml for rod positions A and B, respectively. A similar trend was observed in the moderate (R10C10) disease model where changes of 54 ± 5 ml and 75 ± 10 were noted for rod positions A and B, respectively. A linear regression trend with R^2^ = 1 ± 0.005 in V*_T_* was observed across all simulated lung types, and the slope describes the approximate volume change per cam setting (Figure 4).

**Table 2:** Respiratory parameters for all adjustable-length rod and cam configurations in each of three pulmonary resistance and compliance settings.

		Normal Lung Resistance 5 cmH_2_O.L^-1^.s^-1^, Compliance 50 ml.cmH_2_O^-1^			Mild Disease Resistance 6 cmH_2_O.L^-1^.s^-1^, Compliance 20 ml.cmH_2_O^-1^			Moderate Disease Resistance 10 cmH_2_O.L^-1^.s^-1^, Compliance 10 ml.cmH_2_O^-1^	
Rod Setting	Cam Setting	Pressure (cmH_2_O)	Volume (ml)	Pressure (cmH_2_O)		Volume (ml)	Pressure (cmH_2_O)		Volume (ml)
A	1	3.6	155	7.2		140		12.2	110
A	2	5.3	220	10.5		200		17	160
A	3	7.2	300	14		270		22.5	220
A	4	9	375	17.4		320		28	275
A	5	10.7	450	20.5		370		34.5	325
B	1	5.1	220	8.7		170		14.5	145
B	2	7.6	335	14		270		23	230
B	3	10	420	18		350		32	305
B	4	12.1	530	22		420		38.5	370
B	5	14	610	26		510		N/A	N/A
B	6	15.6	700	N/A		N/A		N/A	N/A

**Figure 5:**
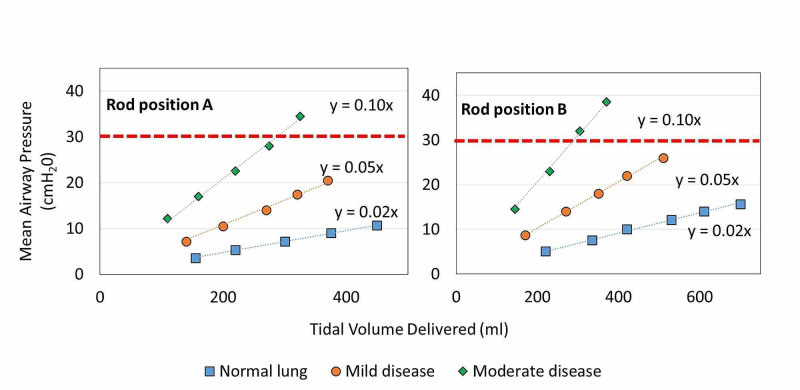
Mean airway pressure measured for simulated patient lung types as a function of tidal volume delivered. Red dashed line denotes maximum airway pressure threshold for ARDS treatment recommendations.

Similar trends were observed in measured P*_aw_* for all three patient models. A linear increase in P*_aw_* (R^2^ = 1) as a function of V*_T_* demonstrates that as V*_T_* increases, a corresponding increase in P*_aw_* was observed (Figure 5) [4].

**Figure 6:**
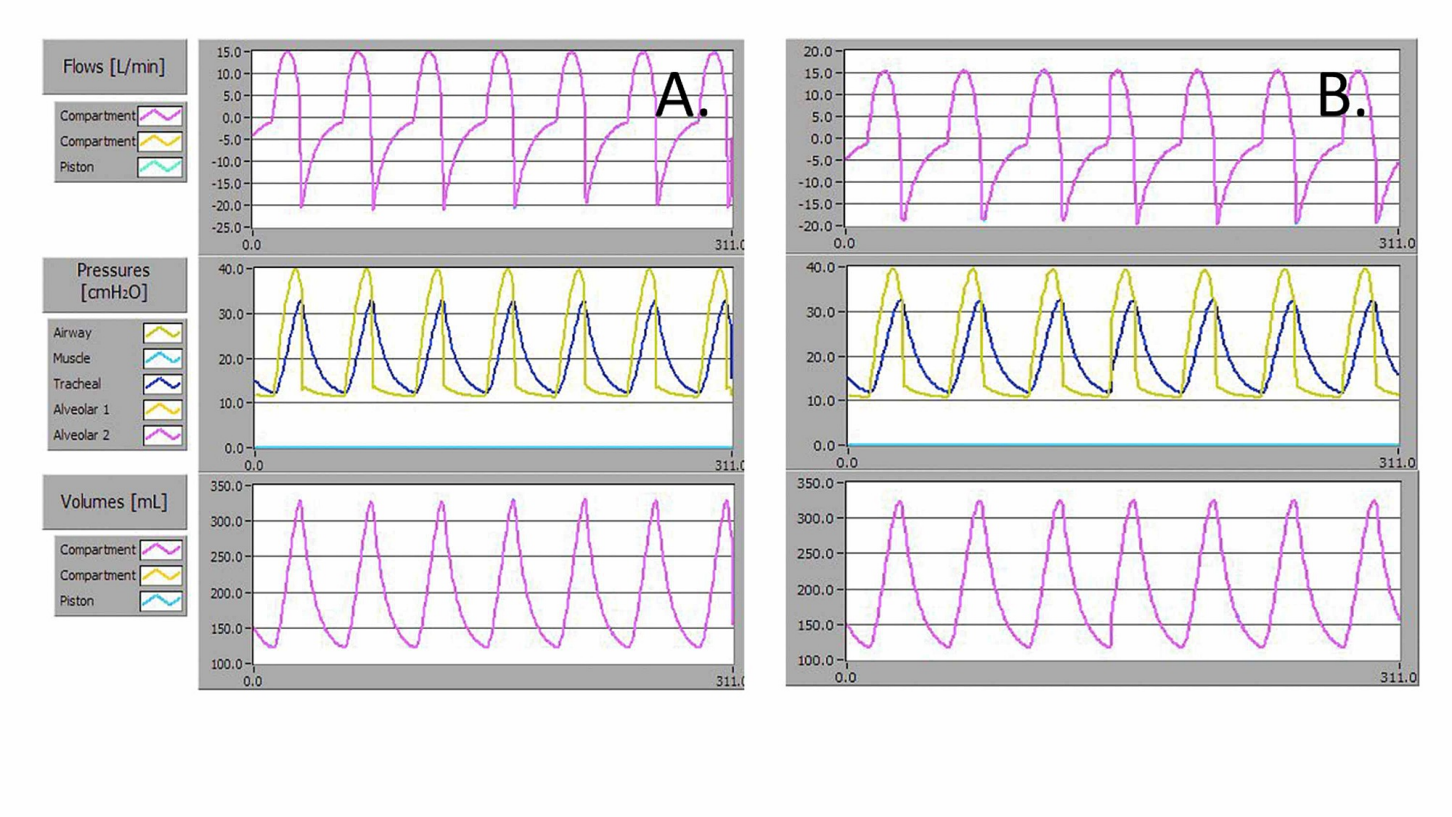
Flow, Paw, and VT waveforms for delivered breaths for a severe disease simulated patient lung model with resistance 50 cmH2O.L-1.s-1 and compliance 10 ml.cmH2O-1 using an AMBU Spur II bag with 10 cmH2O PEEP at A) day zero, and B) after 24 hours of continuous operation.

The AMREV was operated continuously for 24 hours using the severe (R50C10) disease model and positive end-expiratory pressure (PEEP) 10 cmH_2_O to meet ISO 80601-2-80:2018; 201-104 testing requirements [15]. Waveform data before and after this simulated worst-case condition testing was recorded (Figure 6A-B). To meet this requirement, the AMREV was operated with an Ambu Spur II BVM with external PEEP 10, and at setting A3 with a respiratory rate of 20 rpm. The average measured P*_aw_* for 13 breaths at time zero was 39.9 ± 0.1 cmH_2_O and after 24 hours was 39.7 ± 0.1 cmH_2_O. The average V*_T_* delivered over 13 breaths at time zero was 370 ± 1 ml and after 24 hours was 362 ± 1 ml. 

C. Longevity and implementation of AMREV across multiple BVM models

**Figure 7:**
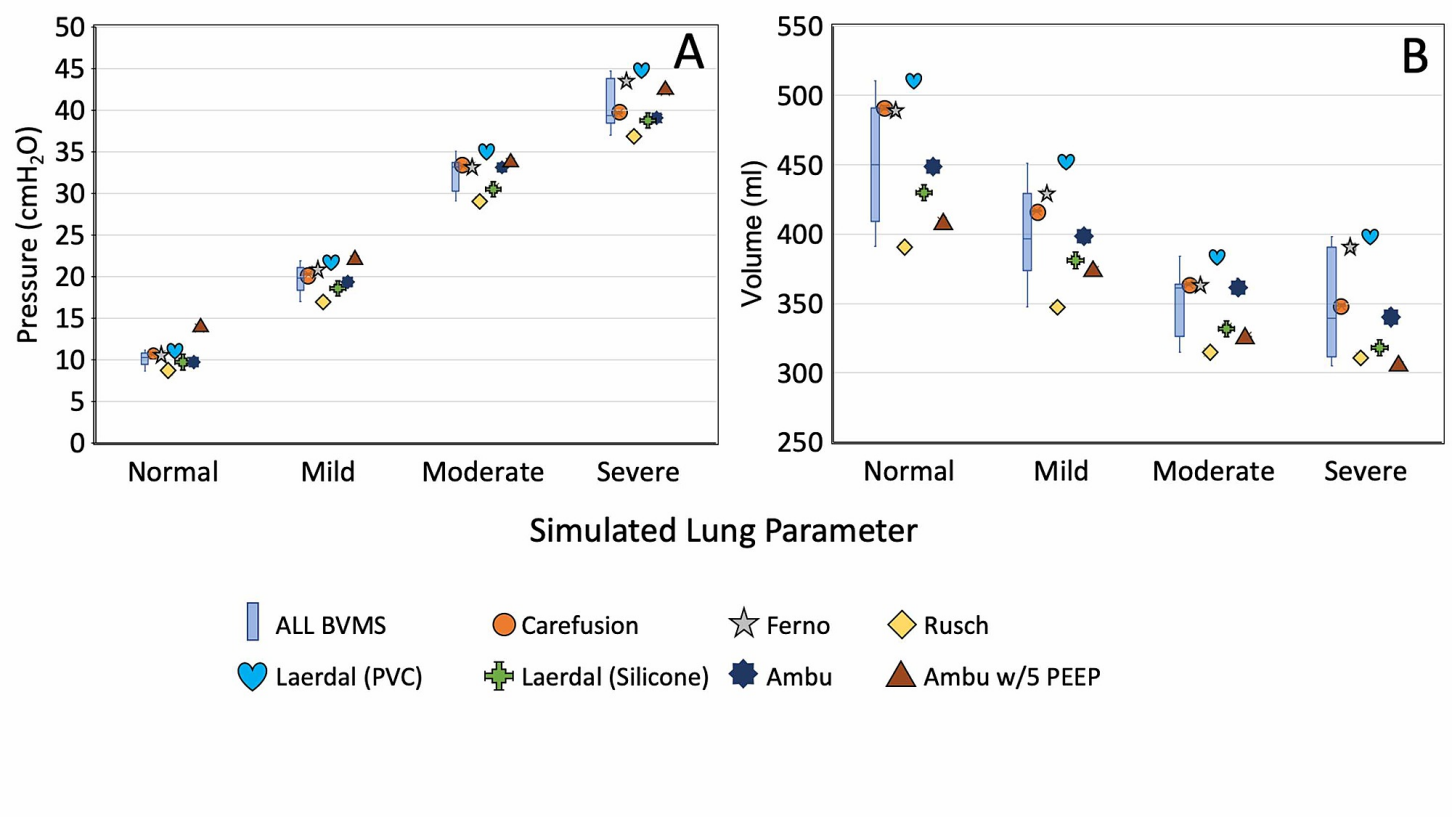
A) Tidal volume VT and B) mean airway pressure Paw measurement, from different BVM models in simulated patient testing with (resistance cmH2O.L-1.s-1, compliance ml.cmH2O-1) of ‘normal’ (5,50), ‘mild’ (6,20), ‘moderate’ (10,10) and ‘severe’ (50,10), respectively.

Widespread use of the AMREV relies on availability and compatibility with commercially-available BVMs. We conducted a study to assess the inherent variability of different BVM models. Seven BVM setups representing six different BVM models and manufacturers were assessed (Figure 2). Two Ambu® bags, one with and one without external PEEP, were independently evaluated. P*_aw_* variation between bags was calculated (Figure 2C). The Ambu® bag with external PEEP was excluded due to baseline P*_aw_* elevation. Variation within each individual bag type in V*_T_* and P*_aw_* was < 0.5% for each BVMs tested, across four simulated lung models. The P*_aw_* for all six bags were 10.1 ± 0.9 cmH_2_O for the normal lung model, 19.7 ± 1.7 cmH_2_O for mild disease, 32.4 ± 2.2 cmH_2_O for moderate disease, and 40.4 ± 3.0 for the severe disease model (Figure 7A). The average V*_T_* for all bags was 460 ± 45 ml for the normal lung model, 404 ± 37 ml for mild disease, 353 ± 25 ml for moderate disease, and 351 ± 36 ml for the severe disease model (Figure 7B). Overall, variation was less than 10% for P*_aw_* and V*_T_* across all bags for each of the four simulated lung models.

**Figure 8:**
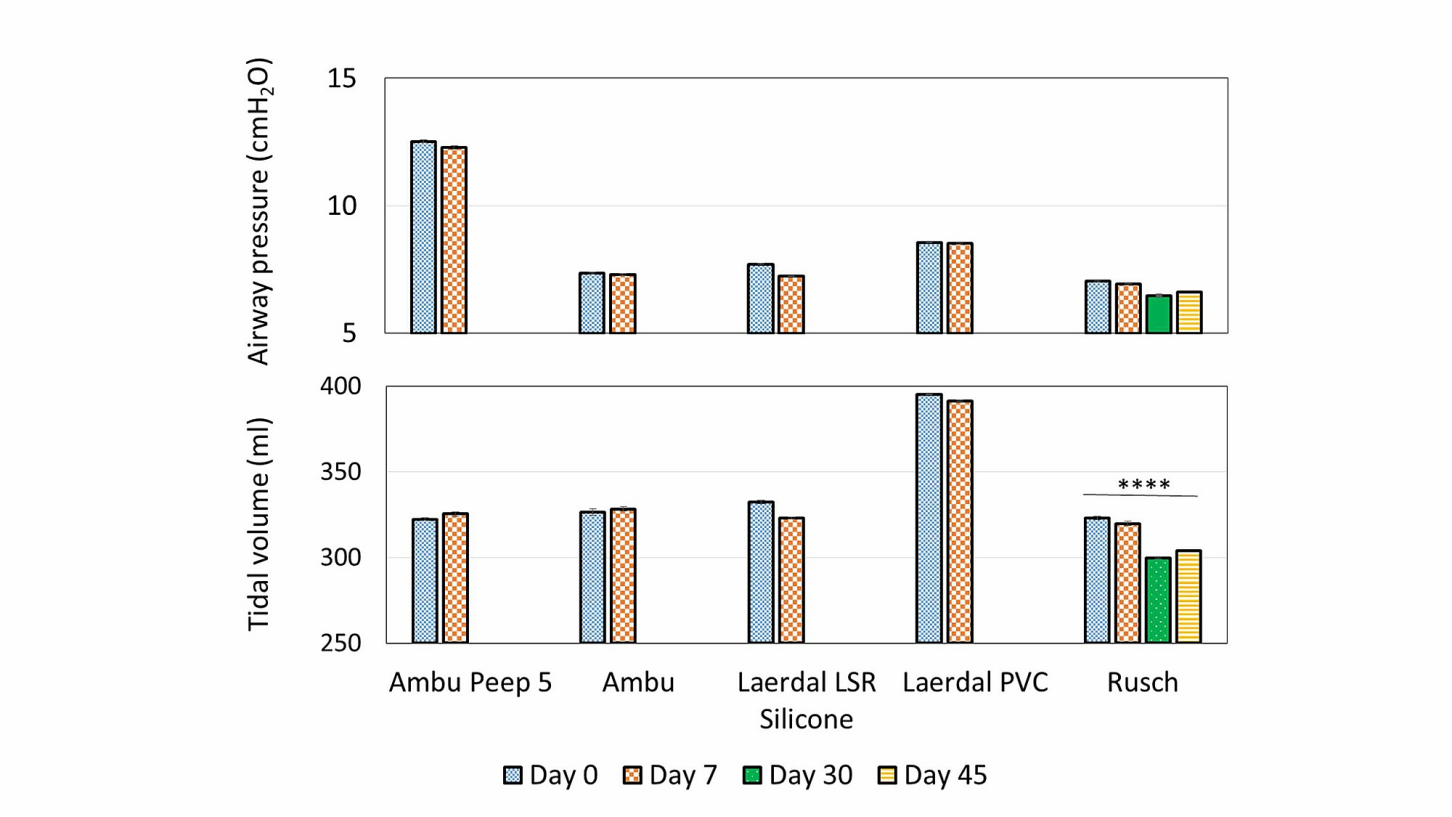
VT and Paw for 5 models of manual resuscitator devices in a 'normal' patient model with resistance = 5 cmH2O.L-1.s-1 and compliance = 50 ml.cmH2O-1 at trial start, after 1 week, 30 days, and 45 days of continuous use. Error bars lie within the column. Ordinary one-way ANOVA P = 0.0001 for Rüsch BVM.

Long-term, multi-day, testing was performed with six BVM devices with settings of compliance 50 ml.cmH_2_O^-1^ and resistance 5 cmH_2_O.L^-1^.s^-1^ to assess for variation within the same BVM (Figure 8). One of the AMREV devices that housed the Carefusion BVM had a component failure on the seventh day and did not have data recorded prior to its failure. The respiratory rate at 75% motor power was 22 ± 2 bpm. V*_T_* and P*_aw_* were independent of the respiratory rate. Data for five BVM/device pairings B-F listed in Table 1 were recorded at seven days. After a one-week course (>230,000 cycles) of continuous use, the V*_T_* variation observed between each bag was less than 1.5% (Table 3). Similar results were observed with P*_aw_* where differences were all within 1%, with one exception; the Laerdal LSR Silicone bag showed variability of 3.2% in P*_aw_* after one week of continued use (Table 4). One device with the Rüsch® BVM (Table 3), completed the seven-day test and continued to 45 days of operation. This BVM showed that V*_T_* and P*_aw_* variation was within 3.8% even over the extended operation.

**Table 3:** Average tidal volume delivered (ml) for each BVM tested, at the start of the extended day trial, and after the completion of seven days of continuous use.

	Ambu PEEP 5 (ml)	Ambu (ml)	Laerdal LSR Silicone (ml)	Laerdal PVC (ml)	Rüsch (ml)
Day 0	322 +/- 1	327 +/- 2	332 +/- 2	395 +/- 0.5	323 +/- 1
Day 7	325 +/- 1	328 +/- 2	323 +/- 1	391 +/- 0.5	320 +/- 1
Day 30					300 +/- 0.4
Day 45					304 +/- 0.4
Percent difference	0.5	0.2	1.4	0.5	3.7

**Table 4:** Average measured peak airway pressures (cmH2O) at the start of the extended day trial and after seven days of continuous use.

	Ambu PEEP 5 (cmH_2_O)	Ambu (cmH_2_O)	Laerdal LSR Silicone (cmH_2_O)	Laerdal PVC (cmH_2_O)	Rüsch (cmH_2_O)
Day 0	12.5	7.3	7.7	8.6	7.0
Day 7	12.3	7.3	7.2	8.5	6.9
Day 30					6.5
Day 45					6.6
Percent difference	1.0	0.3	3.2	0.2	3.8

## Discussion

This study suggests that the use of a BVM-based ventilator system can deliver appropriate respiratory support in the event of a ventilator shortage. The AMREV can be used to support patients with respiratory distress including respiratory failure based on the range of V*_T_*, pressures, and respiratory rates supported. Simulation testing was performed using realistic patient models and transition to real patient care is anticipated based on the study results.

The differences observed in P*_aw_* as a function of V*_T_* delivered were consistent with defined characteristics of pulmonary resistance and compliance (Figure 5). Treatment recommendations for patients with ARDS suggest P*_aw_* should not exceed 30 cmH_2_O. For this device, rod and cam position pairings that produce lower V*_T_* should be used in more severe pulmonary disease to deliver ventilations below this threshold. The device was able to provide safe ventilatory support while not exceeding this P*_aw_* threshold. As external PEEP, provided by existing BVM peripherals, is increased it is important to use lower V*_T_* settings to ensure safe ventilation of lungs with low compliance.

The AMREV demonstrated compatibility with different models of BVM that had diameters ranging from 122-128.5 mm or approximately five inches. These bags are representative of adult BVMs that have an air reservoir of ~1.5 L. While differences between BVMs were identified, overall variation was less than 10% for P*_aw_* and V*_T_* delivered across all bags as assessed over four simulated lung states.

Device life testing demonstrated consistent and predictable delivery of ventilation over an array of patient pulmonary parameters and over time using multiple BVMs. Specifically, six devices were tested over seven days and no failures were identified with the BVM devices or housing structure. Two devices failed at seven days. One of these devices allowed for data collection on the seventh day, however, data was not obtainable from the other device to include in longevity analysis (Tables 3-4). One device continued to function for 45 days with no failures of the BVM, housing, or motor. The failures identified on all devices were related to motor fatigue, with the average device life being 17 days. Motor fatigue was predictable due to increased noise at least 24 hours prior to failure. Future design iterations will incorporate higher-quality motors to improve longevity.

Even with variations in respiratory rate from the PWM setting, respiratory parameters of V*_T_* and P*_aw_* were consistent for each rod and cam setting. Although the device was periodically stopped for testing, this practice is consistent with clinical use. In a clinical setting, a patient may be moved or disconnected from a ventilator to perform testing, thus the study demonstrated that even with minor variations in respiration rate from motor power adjustments during these periods of disconnection and testing, the other respiratory parameters remained consistent.

As described in previous literature, manual BVM compressions,* i.e.* bagging, is variable in rate and V*_T_* delivered [9]. The study findings suggest that the AMREV is able to provide a consistent rate and V*_T_* of respirations and is thus expected to be more reliable and safer than manual bagging. Additionally of interest to medical professionals, the AMREV is adaptable to multiple clinical environments with a housing design and attachment configuration that supports mounting of the device to a bedside IV pole. This design consideration minimizes the distance from the BVM to the endotracheal tube and limits the risk of rebreathing CO_2_.

The study findings suggest that this BVM-based emergency use ventilator can provide consistent positive pressure volume-controlled ventilation over an extended duration until a traditional ventilator becomes available. This device is only intended for use when a traditional ventilator is not available. True shortages may lead to the presented solution being a viable option for some regions during a large-scale health emergency. An imminent predicted need for ventilators resulted in the issuance of an emergency use authorization by the U.S. FDA to increase the availability of devices that provide ventilatory support and even described a new class of ventilation device termed ‘emergency use resuscitator systems’ (EURS) [23]. While these guidelines were established specifically for the COVID-19 pandemic, the knowledge and design innovation obtained from this rapid period of discovery may have lasting effects on future ventilator devices.

## Conclusions

In response to the COVID-19 pandemic that has overwhelmed healthcare systems worldwide, the authors designed, rapidly prototyped, and tested a 3D-printed automated volume-controlled manual-resuscitator based ventilator-alternative. The AMREV was developed to provide respirations with commercially-available BVMs when a ventilator is not available. User-adjustable configurations allow for quick adjustment of tidal volume and respiratory rate and are within the treatment recommendations from the ARDS Network. As the COVID-19 crisis is overwhelming health care systems, leaving some institutions without an adequate supply of ventilators, the AMREV provides a potentially life-saving alternative.

## Figures and Tables

**Figure 9:**
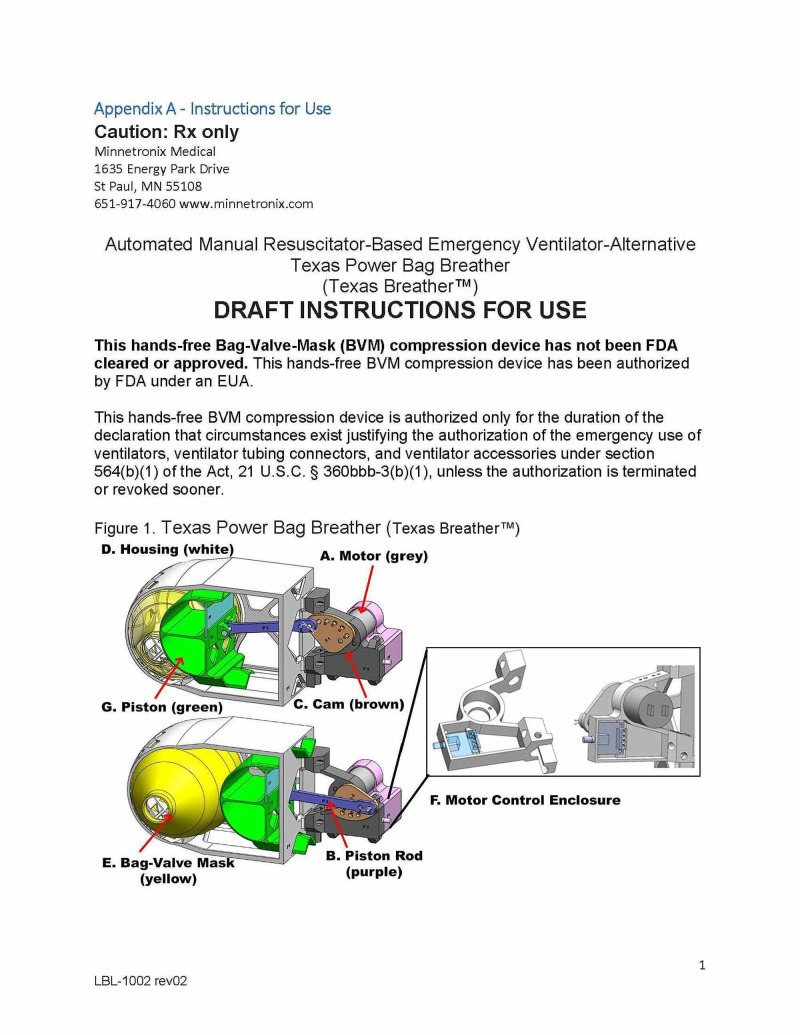
Appendix A.1 Instructions for use

**Figure 10:**
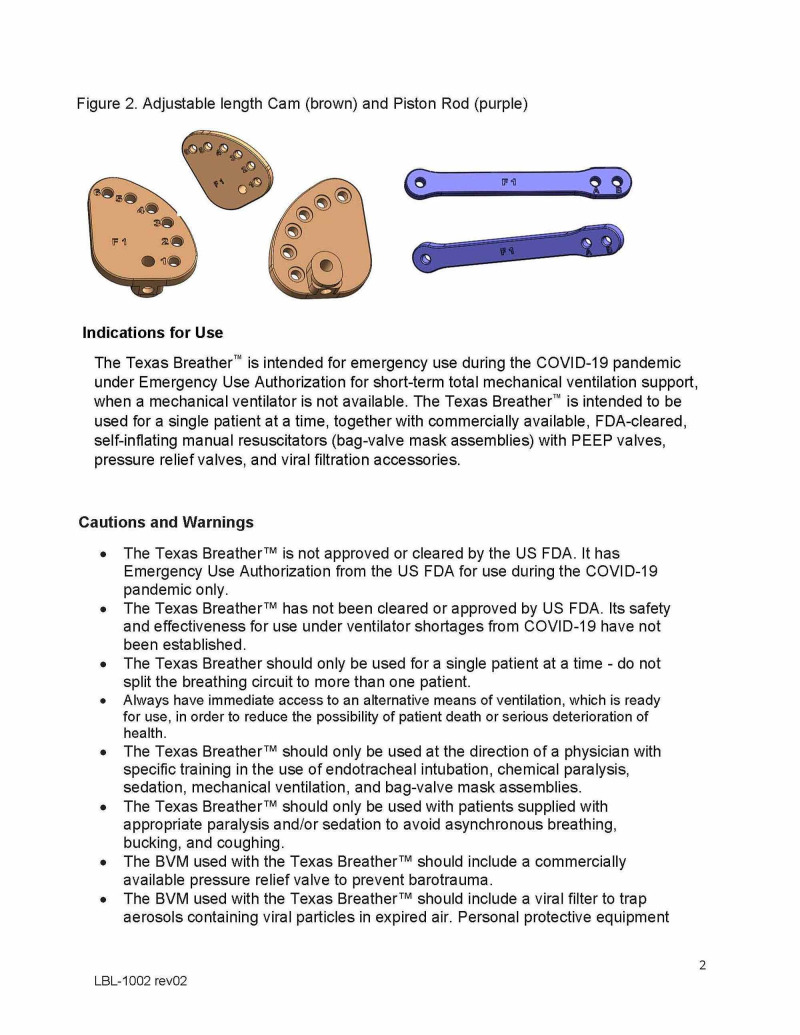
Appendix A.2 Instructions for use

**Figure 11:**
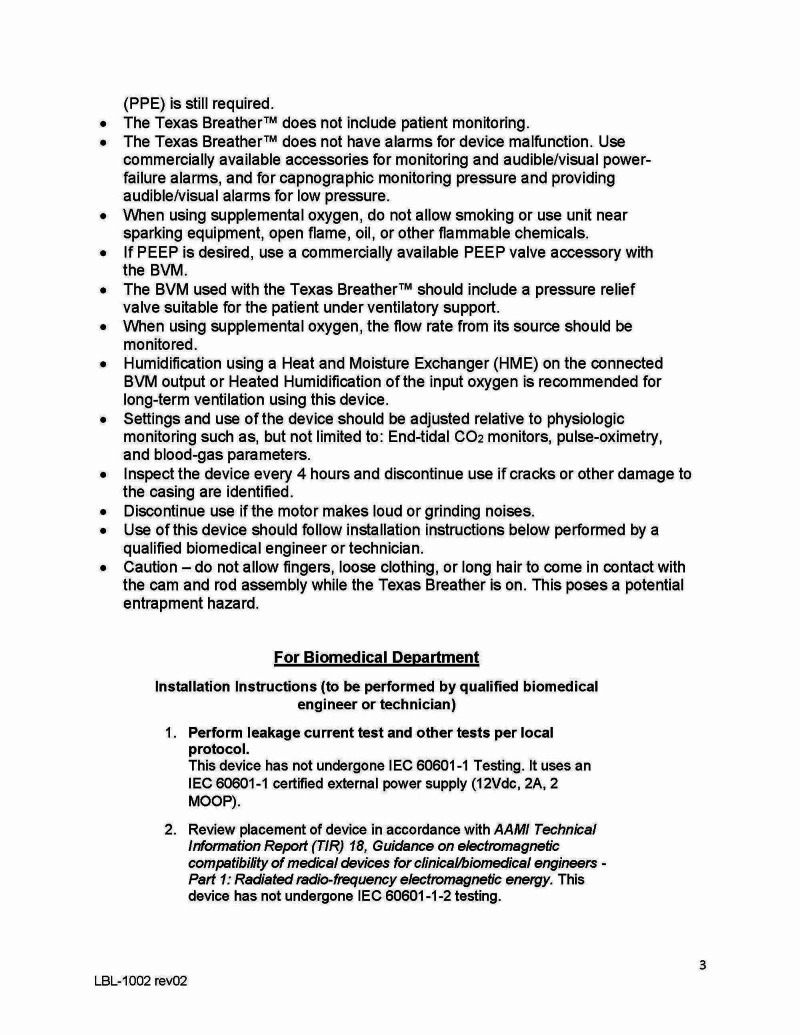
Appendix A.3 Instructions for use

**Figure 12:**
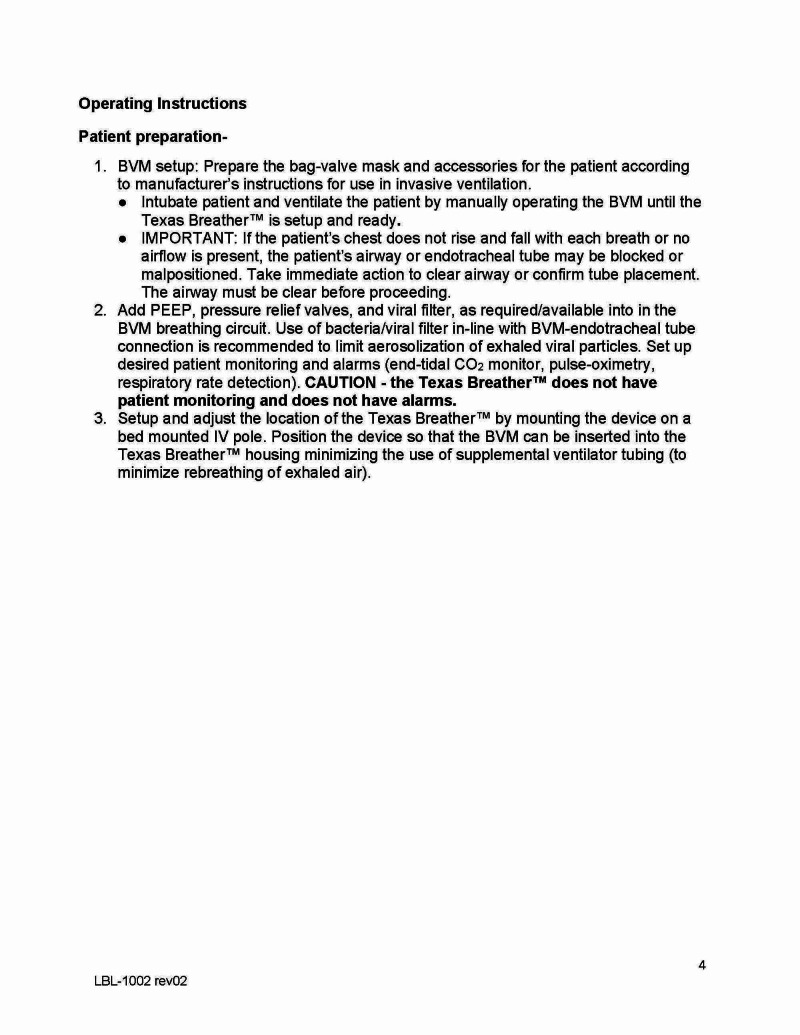
Appendix A.4 Instructions for use

**Figure 13:**
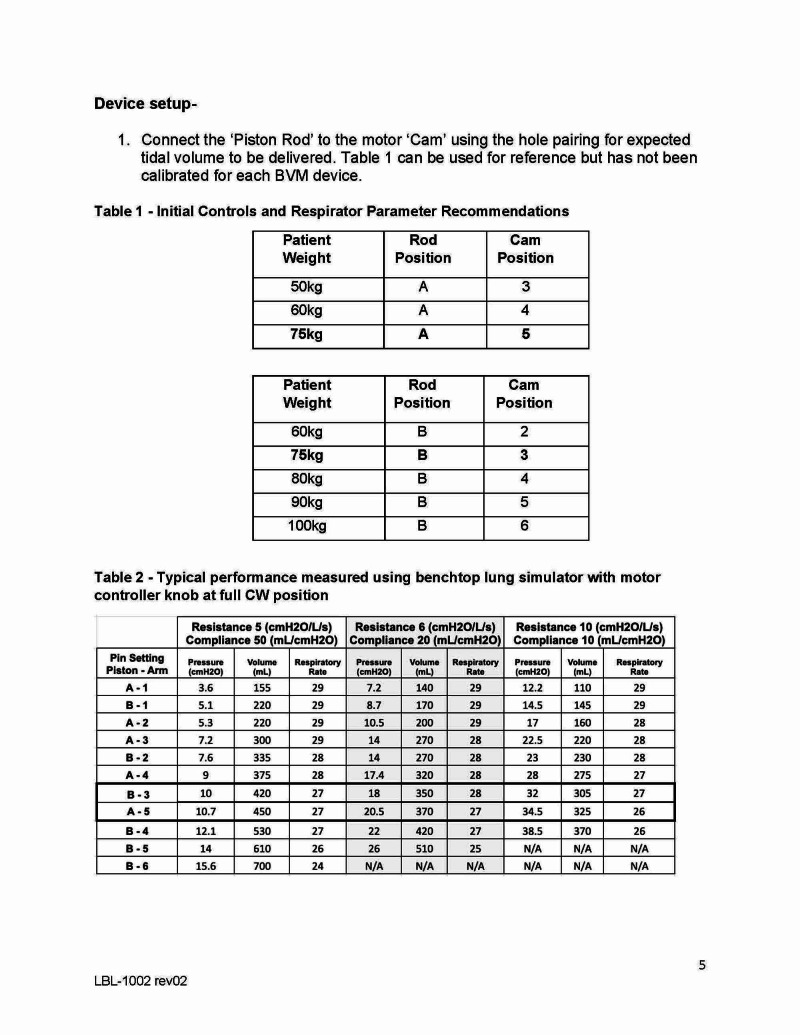
Appendix A.5 Instructions for use

**Figure 14:**
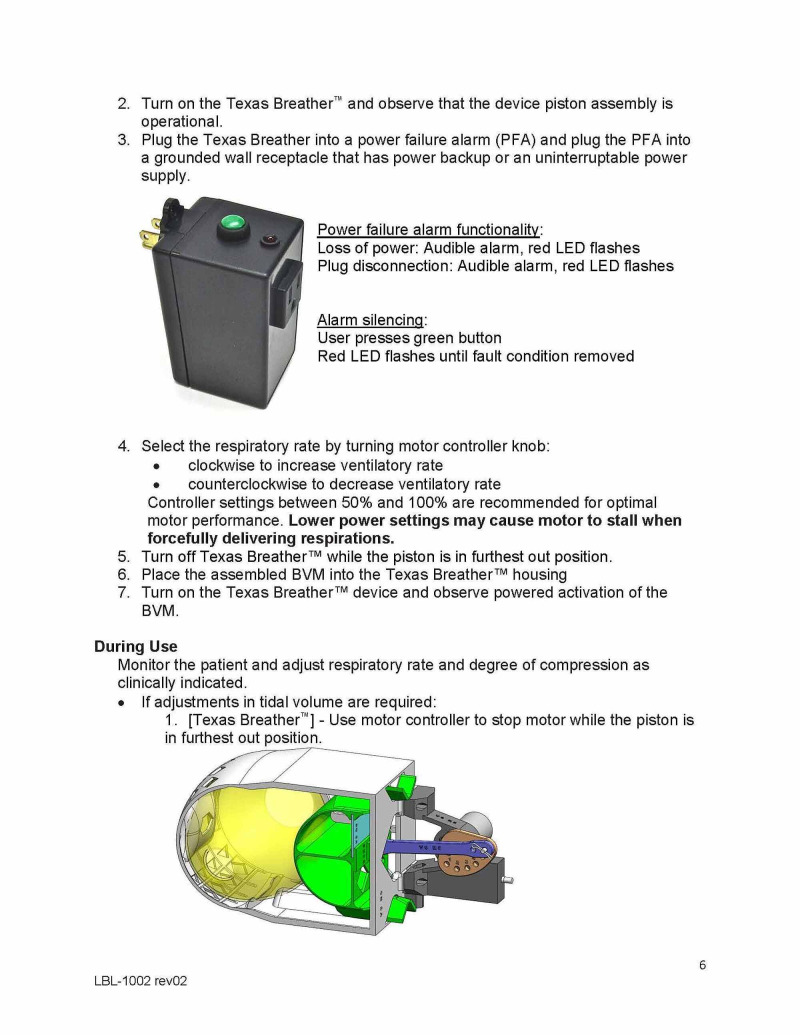
Appendix A.6 Instructions for use

**Figure 15:**
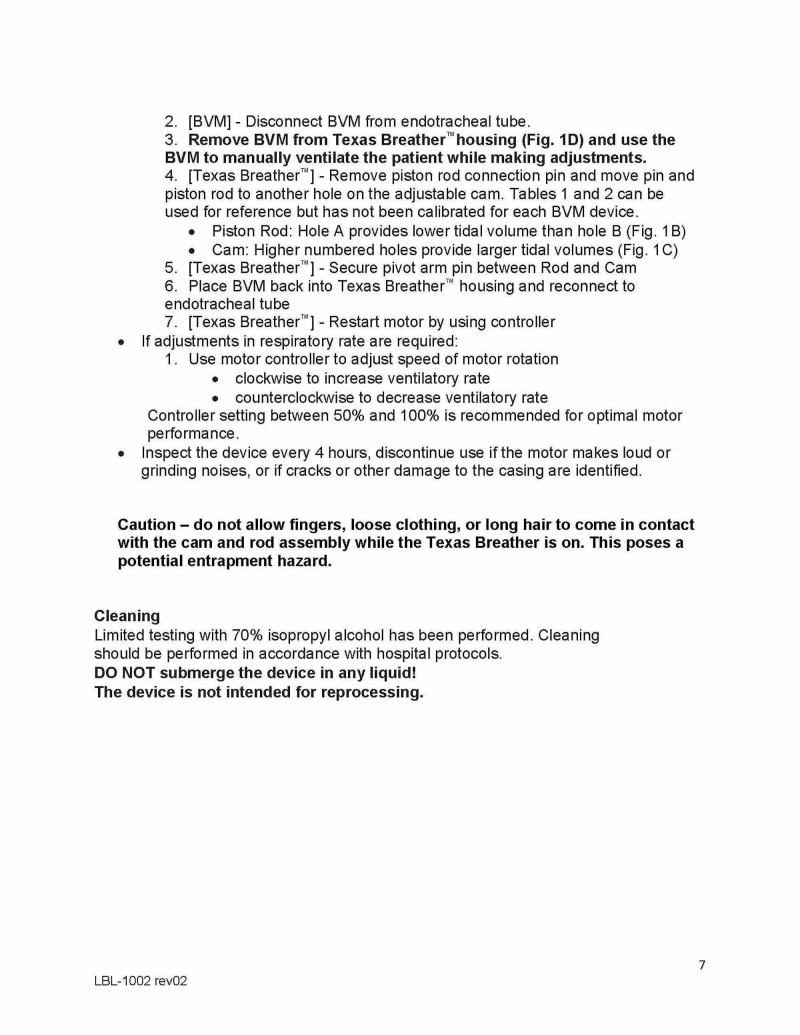
Appendix A.7 Instructions for use

## References

[1] Xu Z, Shi L, Wang Y (2020). Pathological findings of COVID-19 associated with acute respiratory distress syndrome.. Lancet Respir Med.

[2] Gattinoni L, Chiumello D, Caironi P, Busana M, Romitti F, Brazzi L, Camporota L (2020). COVID-19 pneumonia: different respiratory treatments for different phenotypes?. Intensive Care Med.

[3] Siegel Siegel, M.D. and Hyzy, R.C. R.C. (2020). Ventilator management strategies for adults with acute respiratory distress syndrome. Uptodate.com.

[4] Acute Respiratory Distress Syndrome Network, Brower RG, Matthay MA, Morris A, Schoenfeld D, Thompson BT, Wheeler A (2000). Ventilation with lower tidal volumes as compared with traditional tidal volumes for acute lung injury and the acute respiratory distress syndrome. N Engl J Med.

[5] Truog RD, Mitchell C, Daley GQ (2020). The toughest triage—allocating ventilators in a pandemic. N Engl J Med.

[6] Ranney ML, Griffeth V, Jha AK (2020). Critical supply shortages—the need for ventilators and personal protective equipment during the Covid-19 pandemic. N Engl J Med.

[7] Ajao A, Nystrom SV, Koonin LM (2015). Assessing the capacity of the US health care system to use additional mechanical ventilators during a large-scale public health emergency. Disaster Med Public Health Prep.

[8] Khoury A, Sall FS, De Luca A, Pugin A, Pili-Floury S, Pazart L, Capellier G (2016). Evaluation of bag-valve-mask ventilation in manikin studies: what are the current limitations?. Biomed Res Int.

[9] (2020). Openlung emergency medical ventilator. https://gitlab.com/open-source-ventilator/ventilator/OpenLung..

[10] (2020). MPS open-source ventilator. Monolithic power systems, mps open-source ventilator.

[11] (2020). MIT emergency ventilator (e-vent) project. https://emergency-vent.mit.edu/.

[12] Bein T, Grasso S, Moerer O (2016). The standard of care of patients with ARDS: ventilatory settings and rescue therapies for refractory hypoxemia. Intensive Care Med.

[13] Hess DR (2014). Respiratory mechanics in mechanically ventilated patients. Respir Care.

[14] (2020). Food and drug administration, FDA combating covid-19 with medical devices. https://www.fda.gov/media/136702/download.

[15] (2020). ISO, ISO 80601-2-80: 2018, in medical electrical equipment — part 2-80: particular requirements for basic safety and essential performance of ventilatory support equipment for ventilatory insufficiency. https://www.iso.org/standard/68844.html.

[16] (2020). Association for the advancement of medical instrumentaiton (AAMI), emergency use resuscitator system (EURS) design guidance. https://www.aami.org/docs/default-source/standardslibrary/200410_cr503-2020_rev1.pdf.

[17] (2020). The bag II disposable resuscitator. https://www.laerdal.com/us/products/medical-devices/airway-management/the-bag-ii-disposable-resuscitator/..

[18] (2020). CareFusion AirLife® manual self-inflating resuscitation devices. https://www.vyaire.com/products/self-inflating-resuscitation.

[19] (2020). Rusch manual resuscitator bag. Rusch Manual Resuscitator Bag.

[20] (2020). Ambu® SPUR® II - disposable resuscitator. https://www.ambuusa.com/emergency-care-and-training/resuscitators/product/ambu-spur-ii.

[21] Ferno Ferno (2020). Ferno single patient use bvm with medication and monitoring port. https://fernoems.com/oxygen-airway/single-patient-use-bvm.

[22] (2020). Laerdal silicone resuscitators. https://laerdal.com/gb/doc/117/Laerdal-Silicone-Resuscitators-LSR.

[23] Drinker Drinker, P.A. and C.F. McKhann (2020). U.S. Food and drug administration emergency use authorization for ventilators during covid-19 pandemic. JAMA.

